# Phosphodiesterase 5a Signalling in Skeletal Muscle Pathophysiology

**DOI:** 10.3390/ijms24010703

**Published:** 2022-12-31

**Authors:** Valeria De Arcangelis, Luciana De Angelis, Federica Barbagallo, Federica Campolo, Ana Gabriela de Oliveira do Rego, Manuela Pellegrini, Fabio Naro, Mauro Giorgi, Lucia Monaco

**Affiliations:** 1 Neurology Unit Department of Neuroscience, Fondazione Policlinico Univeristario Agostino Gemelli IRCC, 00168 Rome, Italy; 2 Department of Anatomical, Histological, Forensic Medicine and Orthopedics Sciences, Sapienza University, 00161 Rome, Italy; 3 Faculty of Medicine and Surgery, “Kore” University of Enna, 94100 Enna, Italy; 4 Department of Experimental Medicine, Sapienza University, 00161 Rome, Italy; 5 Department of Biology and Biotechnology “C. Darwin”, Sapienza University, 00185 Rome, Italy; 6 Institute of Biochemistry and Cell Biology, IBBC-CNR, 00015 Rome, Italy; 7 Department of Physiology and Pharmacology, Sapienza University, 00185 Rome, Italy

**Keywords:** phosphodiesterases, *Pde5a* isoforms, muscular disorders, atrophy

## Abstract

Phosphodiesterase 5A (PDE5A) is involved in cGMP hydrolysis, regulating many physiological processes. Increased activity of PDE5A has been found in several pathological conditions, and the pharmacological inhibition of PDE5 has been demonstrated to have several therapeutic applications. We have identified the presence of three different *Pde5a* isoforms in cardiomyocytes, and we have found that the expression of specific *Pde5a* isoforms may have a causal role in the onset of pathological responses in these cells. In our previous study, we demonstrated that PDE5A inhibition could ameliorate muscular dystrophy by acting at different levels, as assessed by the altered genomic response of muscular cells following treatment with the PDE5A inhibitor tadalafil. Thus, considering the importance of PDE5A in various pathophysiological conditions, we further investigated the regulation of this enzyme. Here, we analysed the expression of *Pde5a* isoforms in the pathophysiology of skeletal muscle. We found that skeletal muscle tissues and myogenic cells express *Pde5a1* and *Pde5a2* isoforms, and we observed an increased expression of *Pde5a1* in damaged skeletal muscles, while *Pde5a2* levels remained unchanged. We also cloned and characterized the promoters that control the transcription of *Pde5a* isoforms, investigating which of the transcription factors predicted by bioinformatics analysis could be involved in their modulation. In conclusion, we found an overexpression of *Pde5a1* in compromised muscle and identified an involvement of MyoD and Runx1 in *Pde5a1* transcriptional activity.

## 1. Introduction

The second messenger cGMP signalling plays a fundamental role in skeletal and myocardial muscle functions [1,2,3,4]. The intracellular levels of the second messenger cGMP are finely regulated by the rate of synthesis by guanylate cyclase and its degradation by phosphodiesterases (PDEs). PDEs are categorized into 11 families based on sequence homology, sensitivity to inhibitors, and substrate specificity [5,6,7]. These enzymes are ubiquitously distributed in mammalian tissues and interact with signaling molecules to generate macromolecular complexes essential for intracellular signaling pathways that can be altered in various pathological conditions, such as heart diseases, diabetes, pulmonary hypertension, autoimmune diseases, and neurological disorders [8,9,10,11,12,13,14,15,16]. PDE families which specifically hydrolyze cGMP include PDE5A, 6, and 9; other PDEs (PDE1, 2, 3, 10) have as substrate both cAMP and cGMP. The presence of multiple PDEs in the same cell, localized to different subcellular compartments, allows cGMP compartmentalization within the cell [17,18,19].

In skeletal muscle, cells predominantly contain cAMP-specific-PDEs compared with cGMP-specific PDE [20,21,22]. PDE5A is expressed, although at low levels, in skeletal and cardiac muscle cells, and its expression increases under pathological conditions [23,24,25,26]. The important role played by this enzyme is indicated by the protective effects exerted by its inhibitors in several muscular dysfunctions. Beneficial effects of long-term treatments with the PDE5A inhibitor sildenafil were observed following cardiac hypertrophy, diabetic cardiomyopathy, ischemic insult, and doxorubicin cardiotoxicity [27,28,29,30,31]. In addition, the treatment with PDE5A inhibitors (sildenafil or tadalafil) can improve the contractile performance of the dystrophic muscles [32,33,34]. These protective effects are due to both an amelioration of the vascular system by an increased blood flow and a direct effect on the muscular cells. In a preclinical study, we demonstrated that the tadalafil effect acted at different levels, as assessed by the altered genomic response of muscular cells following drug treatment [34]. The main effect of tadalafil was to trigger a metabolic reprogramming of myofibers towards a profile more resistant to contraction-induced damage. In vitro studies confirmed a direct role of PDE5A on cardiac and skeletal muscle cells. In C2C12 myogenic cell lines, the addition of a PDE5A inhibitor improved oxidative metabolism and antioxidant system [35,36] and increased global protein synthesis [34,37].

We identified, in mouse tissues, three splice variants of the *Pde5a* [38]; we also cloned these isoforms in a yeast system and characterized their specific role in the regulation of cellular metabolism [39,40,41]. In vitro studies, in cardiac cells overexpressing murine *Pde5a* isoforms, demonstrated that they have a different subcellular localization, with isoform A1, prevalently cytoplasmic and isoforms A2 and A3, localized both in the cytoplasm and in the nucleus. In addition, the overexpression of the three isoforms increased the percentage of binucleated cells and, to a different extent, cell size, thus suggesting an involvement in the hypertrophic process.

In this study, we further investigated the regulation of this enzyme in several skeletal muscle conditions; moreover, the promoters that control the transcription of the different isoforms were isolated and characterized.

## 2. Results

### 2.1. Pde5a Expression in Murine Skeletal Muscular Cells

**Figure 1:**
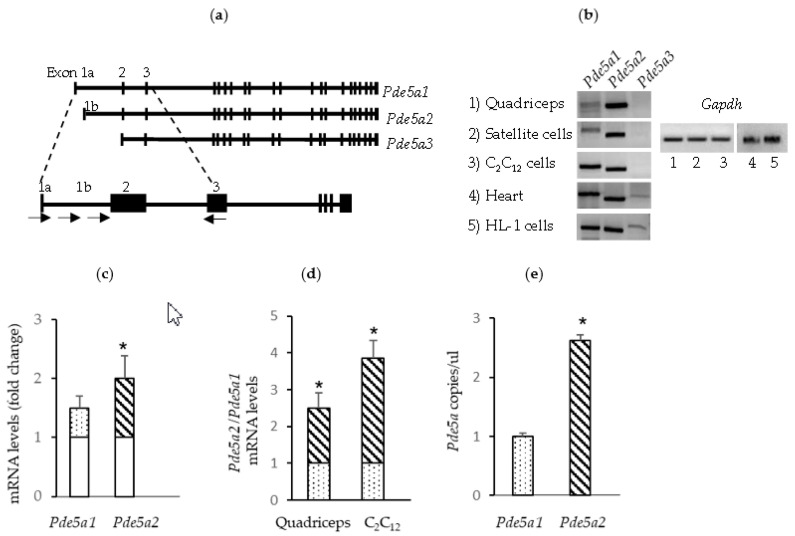
mRNA *Pde5a* isoform expression in murine skeletal and cardiac muscle tissue and cells. (**a**) A schematic representation of *Pde5a* gene showing the three known variants and localization of primers used for PCR; black boxes represent exons. (**b**) RT-PCR analysis performed on RNA isolated from mouse quadriceps tissue, mouse satellite cells, C2C12 myotubes, heart tissue and HL-1 cells. (**c**) *Pde5a1* and *Pde5a2* RNA levels in proliferating and differentiating C2C12 cells measured by RT-qPCR. The mRNA levels in the samples from differentiating C2C12 cells are expressed as the fold change (dotted and striped columns) compared with the samples from proliferating C2C12 (empty columns). (**d**) Relative expression levels of *Pde5a2* (striped columns) isoform compared with *Pde5a1* (dotted columns) in muscle and myogenic cells. (**e**) ddPCR report of RNA analysis from C2C12 cells samples. All values represent the mean ± SD of at least 4 different RNA preparations. * Indicates statistical significance (*t*-test: *p* < 0.05).

We previously reported the expression of different *Pde5a* isoforms in mouse heart tissue [38]. Here, we investigated the presence of these isoforms in skeletal muscle cells. In RT-PCR analysis, forward primers specific for the 5′ ends of the different isoforms of *Pde5a* and located in exon/intron 1 and a reverse primer covering a region present in exon 3 were utilized (Figure 1a). Skeletal muscular tissues, satellite cells, and myogenic C2C12 cells expressed both *Pde5a1* and *Pde5a2* isoforms as well as heart tissue and HL-1, a cardiac cell line (Figure 1b). Isoform *Pde5a3* was not detectable in the myogenic cells’ preparation assays; this isoform is present at low levels in adult heart samples as well as in HL-1 a cardiac cell line (Figure 1b). In C2C12 cells, *Pde5a* expression levels increased during differentiation (Figure 1c) and protein appeared to have both cytoplasmic and nuclear localization in myotubes (Supplementary Figure S1).

Both in skeletal muscles and in C2C12 myotubes, isoform *Pde5a2* was expressed at higher levels compared with isoform *Pde5a1,* as documented by qPCR and droplet digital PCR (ddPCR) (Figure 1d,e).

### 2.2. Pde5a1 Increases in Damaged Skeletal Muscles

**Figure 2:**
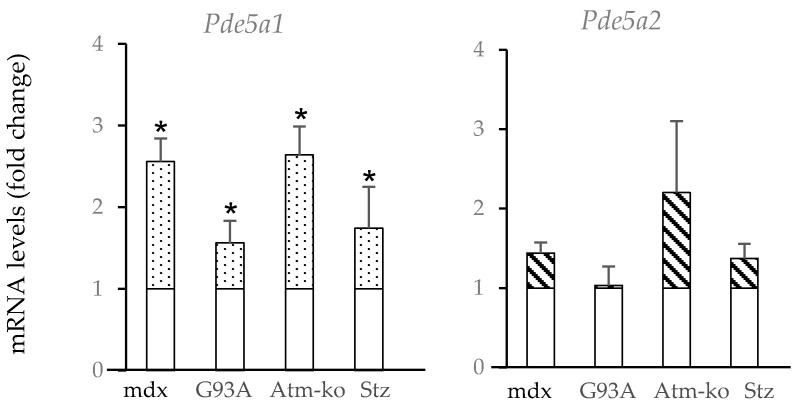
mRNA *Pde5a* isoform expression in skeletal muscle under pathological conditions. mRNA levels of *Pde5a* isoforms were measured by RT-qPCR. mRNA levels in the pathological samples are expressed as the fold change compared with the samples from muscles collected from wild-type mice (empty columns). Relative expression levels of *Pde5a1* (dotted columns) and *Pde5a2* (striped columns) isoform are depicted. All values represent the mean ± SD of at least n = 4 different RNA preparations. * Indicates statistical significance of pathological samples compared with control samples (*t*-test: *p* < 0.05).

To investigate the relative function of the two different *Pde5a* isoforms, we analyzed their expression in different pathological conditions. In muscular tissues from mdx mice, an animal model of Duchenne muscular dystrophy, increased PDE5 activity was observed compared with control tissues [23]. In skeletal muscles from these mice, at an age of 3–4 weeks, when the muscle pathology reaches a peak, we observed a high increase in the *Pde5a1* isoform and to a lesser extent in the *Pde5a2* isoform (Figure 2).

Increased levels of Pde5a1 mRNA were also present in the skeletal muscle of Sod-G93A mice, reflecting a model of neuromuscular disorder, such as ALS (amyotrophic lateral sclerosis) [42], in which skeletal muscles developed progressive muscle atrophy and fibrosis. In other conditions of atrophy, such as muscles from mice lacking ataxia telangiectasia, Atm-ko [43], and in muscles from streptozotocin-induced diabetes mice (Stz) (Figure 2), an upregulation of *Pde5a1* was observed. Concerning the *Pde5a2* isoform RNA levels, no significant changes occurred in the different samples tested.

### 2.3. Promoter Analysis

We then focused to identify the promoter regions of the murine *Pde5a1* and *Pde5a2* isoforms. To this regard, we retrieved the mouse *Pde5a* gene sequence from GenBank and designed primers to amplify regions of approximately 1300 bp upstream from the start codon of *Pde5a1* and *Pde5a2* isoforms (Supplementary Figure S2). Fragments were subcloned in the pGl3 basic vector and then assayed for promoter activity. In initial experiments, the mouse fibroblast NIH-3T3 cells were transiently transfected with these constructs (pGl3-*Pde5a1* and pGl3-*Pde5a2*) and luciferase activity was measured.

**Figure 3:**
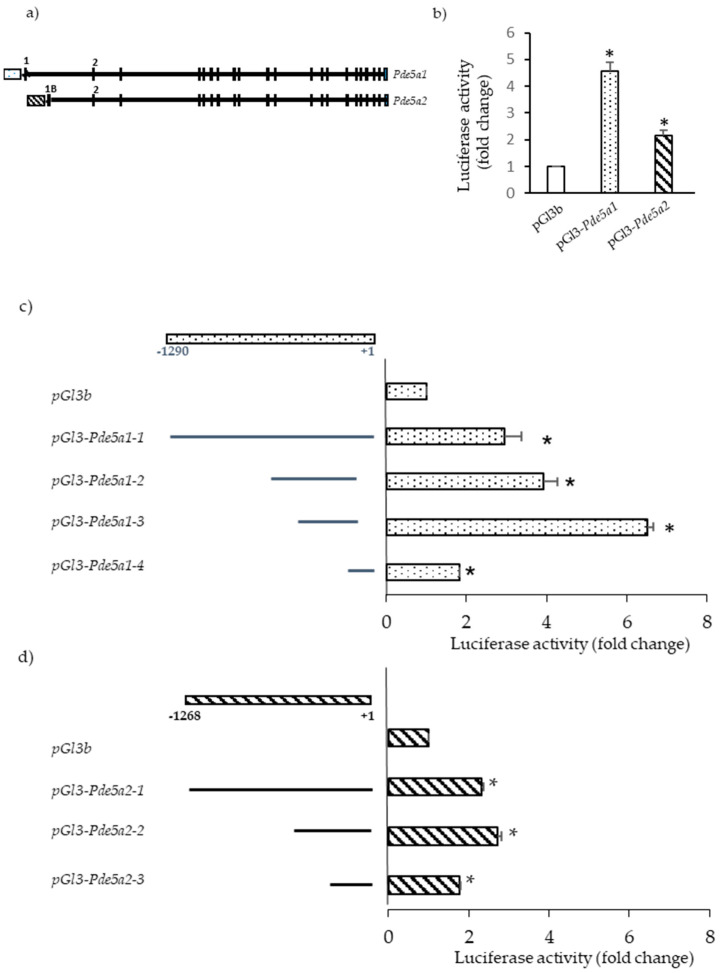
Analysis of *Pde5a1* and *Pde5a2* promoter activity. (**a**) Scheme of *Pde5a* gene organization; dotted and striped boxes represent the putative promoter regions. (**b**) NIH-3T3 cells were transiently transfected with each of the plasmids containing the firefly luciferase reporter under the control of *Pde5a1 and Pde5a2* promoter (**b**) or several fragments of these promoters (**c**,**d**). Results are expressed as the fold change activity relative to that observed with the pGl3 basic empty plasmid. Values represent the mean ± SD of at least n = 4 different experiments performed in triplicate. * Indicates statistical significance of promoter constructs with respect to empty vector (*t*-test: *p* < 0.05).

The result of this assay confirmed that the selected sequences contained promoter activity (Figure 3b). To identify the minimal DNA region still retaining activity, a series of truncated DNA fragments were subcloned in the pGl3 basic vector and tested in NIH-3T3 cells.

Construct pGl3-*Pde5a1*-*3*, covering the region from −375 to −36 from the start codon, possessed the strongest promoter activity, most likely for the presence of sequences target of positive transcription regulators. The promoter segments pGl3-*Pde5a1*-*1* (from −1230 to the start codon) and pGl3-*Pde5a1*-*2* (from −555 to −63 from the start codon) presented reduced activity compared with segment *Pde5a1-3*; this might be due to the presence of sequences, upstream −375 bp, that could be negatively modulated by specific transcription factors.

The *Pde5a2* DNA fragment with the highest luciferase activity included the region 670 bp upstream the starting ATG (*Pde5a2*-2, Figure 3d) while the minimal promoter region of *Pde5a2* was identified in the 400 bp region upstream the first ATG (pGl3-*Pde5a2-3*).

Further analyses in skeletal muscle (C2C12 cells) and cardiac cells (HL-1 cells) confirmed the results obtained in NIH-3T3 cells [44] (data not shown).

### 2.4. Transcription Factors Regulating Pde5a Promoters

Next, several databases were utilized to identify potential transcription factor binding sites. This analysis revealed the presence, in both *Pde5a1* and *Pde5a2* promoters, of numerous putative binding sites for cAMP response element-binding protein (CREB) and SP1. However, luciferase activity in muscle cells transfected with these promoters was only slightly increased following treatment with agents raising cAMP [44] (data not shown).

Other transcription factors with a high score of binding on the *Pde5a1* promoter include MYOD, RUNX1, EGR1, and ATF3.

**Figure 4:**
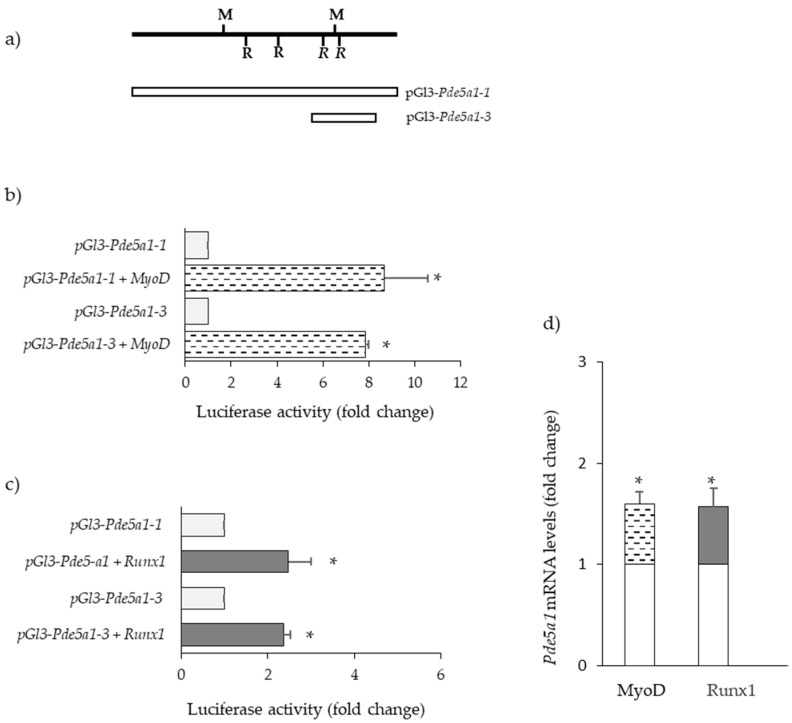
Modulation of *Pde5a1* by MYOD and RUNX1. (**a**) Scheme of *Pde5A* promoter constructs transfected in cells. C2C12 cells were transiently co-transfected with a plasmid expressing MyoD (**b**) or Runx1 (**c**) and the different *Pde5a1* promoter constructs. Results are the means of at least three different experiments performed in triplicate and expressed as the fold change activity relative to that observed in cells not overexpressing MyoD or Runx1. (**d**) C2C12 cells were transiently transfected with a plasmid expressing MyoD or Runx1 and the levels of *Pde5a1* RNA were measured by RT-qPCR. Relative expression of *Pde5a1* in cells overexpressing MYOD (dotted column) or RUNX1 (grey column) are expressed as the fold change compared to untreated cells (empty columns) All values represent the mean ± SD of n = 3 different RNA preparations. * Indicates statistical significance by the *t*-test (*p* < 0.05).

The presence of a binding site for MYOD may explain the higher levels of *Pde5a1* in differentiated C2C12 cells compared with the cell under proliferative conditions. Indeed, we found that the luciferase activities of different *Pde5a1* promoter constructs were higher in co-transfection experiments of C2C12 cells with a plasmid expressing MYOD (Figure 4b).

In myogenic cells, the overexpression of RUNX1 and enhanced *Pde5a1* transcriptional activity were detected (Figure 4c). In line with promoter studies, increased levels of *Pde5a1* RNA were detected in C2C12 cells overexpressing MyoD and Runx1 (Figure 4d).

**Figure 5:**
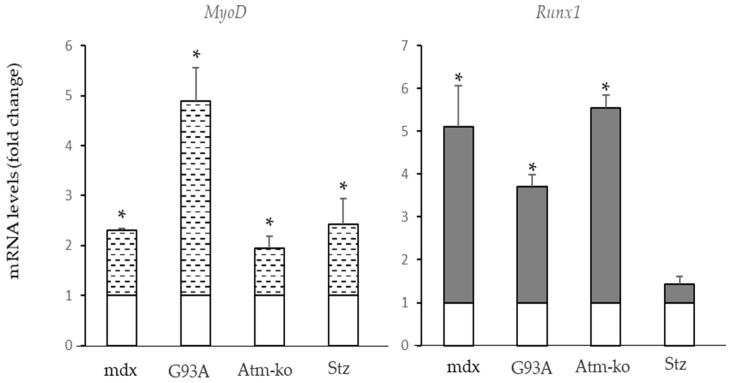
*MyoD* and *Runx1* mRNA levels in skeletal muscle under pathophysiological conditions. The mRNA levels of *MyoD* and *Runx1* were measured by RT-qPCR. mRNA levels in the samples (dotted or grey columns) are expressed as the fold change compared with the samples from muscle from wild-type mice (empty columns). All values represent the mean ± SD of n = 3 different RNA preparations. * Indicates statistical significance of pathological samples compared with control samples (*t*-test: *p* < 0.05).

Data from the literature indicate an increased expression of Runx1 and MyoD in several pathophysiological conditions [45,46,47]. We tested *MyoD* and *Runx1* expression in muscle samples from different conditions. We observed that in the previous models of muscle atrophy/fibrosis which presented an increased expression of *Pde5a1*, increased levels of both *MyoD* and *Runx1* were also detected (Figure 5).

Transcription factors binding sites for Atf3 and Egr1 were present on the *Pde5-a1* promoter, but no modulation of the promoter activity was observed in cells overexpressing these two transcription factors [44](data not shown).

*Pde5a2* is expressed at higher levels compared with isoform *Pde5a1*; however, this isoform is not significantly modulated in the skeletal muscle pathological samples analyzed. The bioinformatics analysis of the *Pde5a2* promoter revealed the presence of several potential transcription factor recognition sites such as AP1, SP1, GATA, SRY, and LYF1/Ikaros. Interestingly, one putative binding site for Myogenin and RUNX1 was also present on *Pde5a2*. However, just a slight increase in *Pde5a2* promoter activity was observed in the presence of MYOD and RUNX1, but no significant increase in *Pde5a2* RNA level was detected [44](data not shown).

## 3. Discussion

Here, we characterized the expression and regulation of *Pde5a* isoforms in skeletal muscle cells. Both *Pde5a1* and *Pde5a2* isoforms are expressed in murine muscular tissues and muscular cell lines. Isoform *Pde5a2* is expressed at higher levels compared with isoform *Pde5a1*. In several human and rodent tissues, a third isoform, *Pde5a3* has been identified, which lacks the 5′ amino regions of the other two isoforms and starts from an ATG present in the second exon common to all the isoforms [38]. This isoform was not detectable in none of the murine skeletal muscle cells analyzed; however, *Pde5a3* is clearly measurable in an embryonic mouse heart, decreases during development, and is present at low levels in a normal adult heart [48].

The behaviour of the two murine *Pde5a* isoforms was analysed in distinct muscular pathological insults. In muscular models characterized by atrophy and fibrosis, such as dystrophic mdx muscles or muscles from SOD1-G93A mice, a transgenic model of ALS (amyotrophic lateral sclerosis) and an increased expression of *Pde5a1* were observed.

Impaired expression of this enzyme was also observed in other conditions where a reduction in muscular mass was present, which is in a condition of cachexia and in a situation of diabetes [43,49,50]. Although isoform *Pde5a2* levels are higher than isoform *Pde5a1*, it seems to be stable in the different conditions examined.

We investigated which factors could regulate the expression of these *Pde5a* isoforms in muscle tissues in pathophysiological conditions. Bioinformatics analysis carried out on multiple databases indicates the presence of several putative binding sites for transcription factors in the two different promoter regions. We did not identify a strong modulation of the cyclic nucleotide signaling on the *Pde51a* promoter activity. We also checked a series of potential regulators of the *Pde5a1* promoter. Among them, we found that only MyoD and RUNX1 are able to influence the transcription of murine *Pde5a1.*

MYOD belongs to the family of myogenic regulatory factors (MRFs) which are implicated in the process of muscle regeneration, and it is essential for the initiation of myoblasts differentiation [51,52]. Its expression increases and remains elevated for the entire regenerative process and drops only at the end of the process. High levels of MyoD have been detected in a different type of muscle damage, such as dystrophy or insult by denervation as in ALS [45,46]. In this latter condition, it has been observed an acceleration of disease progression following MyoD gene transfer [53]. We demonstrated increased transcriptional activity of *Pde5a1* in cells overexpressing MYOD. In addition, we confirmed increased levels of *MyoD* in the different conditions of muscle damage examined.

RUNX1/AML1 is a transcription factor initially identified as being implicated in hematopoietic development and later found to be involved in the regulation of several cellular pathways and systems [54]. In normal conditions, this transcription factor is not expressed in skeletal muscle, but its expression increases in muscle affected by dystrophy, denervation, and age-related muscle loss [47,55,56,57]; it is also induced in several types of cardiac diseases, such as ischaemia, diabetic cardiomyopathy, pressure overload, and dilated cardiomyopathy [58]. It has been suggested that the increased expression of RUNX1 may function to protect myofibers from severe atrophy; in mice with conditional deficiency of RUNX1, the disorganization of myofibrils and autophagy has been observed [59]. Interestingly, it has been reported that this transcription factor is activated in response to muscle damage and works together with MYOD and AP-1/c-Jun transcription factors to carry on the transcription program of muscle regeneration following injury [60].

In the different compromised muscle samples assayed, a raise in *Runx1* levels was detected, thus confirming the involvement of these transcription factors in *Pde5a1* regulation.

The skeletal muscular pathological conditions we investigated point to a role of PDE5A in the atrophy process, that is, an increased expression of this enzyme in conditions with reduced muscular mass. Myostatin is a negative regulator of skeletal mass and in several myostatin knockout (ko) animals, better skeletal muscle development occurs [61]. Recently Gu et al. [62] reported a significant decrease in *Pde5a* expression in the heart from myostatin ko cattle. In addition, these mutant animals presented a high skeletal muscle mass compared with wild-type animals. These results suggest a negative correlation between *Pde5a* expression and muscle mass.

In the last few years, increasing evidence documented the important role played by the PDE family in pathophysiology. Several recent studies support the involvement of PDE enzymes in disorders affecting movement, such as rare forms of Parkinson’s and chorea [11]. Up to now, rare disorders have been detected to be due to germline mutations in the human PDE genes [63]. As far as PDE5, a recent paper documented the identification of a PDE5A variant implicated in coronary artery disease [64]. No observations were reported about the skeletal muscle system.

In conclusion, our data indicate the presence of two isoforms, Pde5a1 and Pde5a2, in murine skeletal muscle cells and an increased expression of *Pde5a1* isoform in compromised skeletal muscles, most likely due to the increased expression of MYOD and RUNX1. Of course, an in vivo genomic analysis of skeletal tissues will be necessary to confirm these data and to identify all other effective regulator molecules. To this purpose, enchip analysis through the innovative technology CRISPR/Cas9 coupled to mass spectrometry could be applied [65,66]. This experimental approach will isolate and identify all the different components interacting with the *Pde5a*1 promoter. Such findings could be useful to identify new putative therapeutic targets for PDE5A-related diseases.

## 4. Materials and Methods

### 4.1. Animals

All procedures involving mice were carried out in accordance with the ethical guidelines for animal care of the European Community Council (directive 2010/63EU), and the studies were approved by the Sapienza University’s Animal Research Ethics Committee and by the Italian Ministry of Health.

Mice were housed under a 12 h light-dark schedule at a constant temperature and with food and water ad libitum. The animals were sacrificed by carbon dioxide asphyxiation. After the animals were sacrificed, skeletal and cardiac muscle tissues were collected and frozen in liquid nitrogen. All the samples were stored at −80 °C.

The C57BL/6J mice and the mdx mice were from Charles River Laboratories Italia s.r.l.-Calco, Lecco, Italy. hSOD1(G93A) mice were obtained from Jackson Laboratory (Bar Harbor, ME, USA). A murine model of ataxia telangiectasia, Atm-ko mice were generated, as previously described [43]. Diabetes was induced in mice by using a single intraperitoneally injection of streptozotocin (Stz; Sigma–Aldrich, Saint Louis, MO, USA) at a dose of 150 mg/Kg, and then, mice were sacrificed after 4 weeks [67].

### 4.2. Cell Cultures

Murine NIH-3T3 cells and myogenic cell line C2C12 were purchased from ATCC (Manassas, VA, USA) and cultured in Dulbecco’s modified Eagle’s medium (DMEM) supplemented with 10% fetal bovine serum (Thermo Fisher Scientific, Waltham, MA USA). To induce myogenic cells to differentiate, the cells were switched to a low serum-containing medium (DMEM plus 2% horse serum) for 5–7 days. HL-1 cardiac cell lines were from Sigma Aldrich (Saint Louis, MO, USA) and cultured in a Claycomb medium (Sigma Aldrich, Saint Louis, MO, USA) supplemented with 10% *v*/*v* FBS, 0.2 mM norepinephrine, 2 mM L-glutamine, 1 U/mL penicillin, and 1 μg/mL streptomycin solution (all from Sigma-Aldrich, Saint Louis, MO, USA).

### 4.3. Cell Immunofluorescence

Proliferating and differentiated C2C12 were fixed with ice-cold 4% paraformaldehyde (Sigma Aldrich, Saint Louis, MO, USA) in Phosphate Buffered Saline (PBS, Sigma-Aldrich, Saint Louis, MO, USA) and incubated for 10 min at 4 °C, washed two times with PBS to remove any paraformaldehyde residue and permeabilized with 0.1% *v*/*v* Triton X-100 (Sigma-Aldrich, Saint Louis, MO, USA) for 10 min at room temperature (RT). Cells were then washed with PBS and incubated for 1 h in 3% *w*/*v* Bovine Serum Albumin (BSA, (Sigma-Aldrich, Saint Louis, MO, USA) at room temperature. Rabbit polyclonal PDE5A antibody (Abcam, Cambridge, UK) was diluted in 1% *w*/*v* BSA and left overnight at 4 °C. After three washings in PBS, cells were incubated with the appropriate secondary antibody fluorescently labeled (fluorescein isothiocyanate, FITC; Thermo Fisher Scientific, Waltham, MA, USA) for 1 h at RT. The labeling of actin microfilaments was performed by staining with Phalloidib-tetramethylrhodamine B isothiocyanate (TRICT, Sigma Aldrich, Saint Louis, MO, USA). Following several washes, cells were counterstained with DAPI and mounted with Vectashield antifade mounting medium (Vector laboratories, Newark, CA, USA). Immunostained samples were examined by a Zeiss Axioskop 2 plus fluorescence microscope; images were acquired by Zeiss AxioCam using the Zen software blue edition (Carl Zeiss Microscopy GmbH, Jena, Germany).

### 4.4. RNA Preparation and Analysis

RNA was isolated from tissues and cells using Trizol reagent (Thermo Fisher Scientific, Waltham, MA, USA) according to the manufacturer’s instructions. Before PCR analysis, a DNase treatment was performed to remove contaminating genomic DNA.

The cDNA was prepared using the High-Capacity cDNA Reverse Transcription kit (Thermo Fisher Scientific, Waltham, MA USA).

PCR analysis was performed with the Dream Taq PCR master mix (Thermo Fisher Scientific, Waltham, MA, USA) following the manufacturer’s protocol. Couples of primers were designed based on the cDNA and genomic sequence and to encompass introns (Table 1).

Quantitative reverse transcription PCR (RT-qPCR) was performed using the BioRad real-time PCR system and reagents. For the quantification analysis, the comparative threshold cycle (Ct) method was used. The Ct values of each gene were normalized to the Ct value of Gapdh in the same RNA sample. The gene expression levels were evaluated by fold change using the equation 2^−ddCt^.

Droplet digital PCR (ddPCR) was performed using BioRad QX100 System (Bio-Rad Laboratories, Hercules, CA, USA) following the manufacturer’s protocol. Amplification was carried out using 1 μL of cDNA template, and 1 μL probe: Gapdh (Applied Biosystems, Thermo Fisher Scientific, Waltham, MA, USA; ref Mm99999915_g1 cat 1645216); Pde5a isoforms (IDT, Coralville, IO, USA; Pde451: ref 65088966 cat 113886671; Pde5a2 ref 65088970 cat113886672) and ddPCR supermix (Bio-Rad Laboratories, Hercules, CA, USA). The PCR mixture was loaded in a disposable droplet generator to create oil-emulsion droplets and transferred to a 96-well PCR plate, sealed, and quantified at the endpoint in a QX200 Droplet Reader System using QuantaSoft software (Bio-Rad Laboratories, Hercules, CA, USA). The fraction of positive droplets was quantified assuming a Poisson distribution. Samples were performed in triplicate, and concentration results were expressed as the number of copies of each transcript/μL sample.

The primers used in qPCR assays are reported in Table 1. Primers efficiency was calculated using the serial dilution method.

### 4.5. Vector Constructs

The promoter regions were synthesized by PCR from a mouse embryonic genomic library. The genomic sequence of PDE5 was retrieved from GenBank. Based on this sequence, oligonucleotide couples were designed to amplify a 1,3 kb region upstream of the first ATG of *Pde5a1* and *Pde5a2*. The PCR products were cloned into a PCR-TOPO vector (Thermo Fisher Scientific, Waltham, MA, USA), sequenced and, subsequently, subcloned into a luciferase reporter plasmid, pGL3basic (Promega, Madison, WI, USA) for luciferase assay. The different short luciferase constructs were generated by PCR or restriction enzyme digestions starting the long construct. Oligonucleotide primers are listed in Table 1. Expression vectors coding for MyoD and Runx1 were purchased from Addgene (Watertown, MA, USA).

### 4.6. Transfection and Luciferase Assays

Transfection was performed using the Lipofectamine 2000 (NIH-3T3 cells) or Lipofectamine 3000 reagent (C2C12) (Thermo Fisher Scientific, Waltham, MA, USA) according to the manufacturer’s protocol.

To assess luciferase activity, NIH-3T3 cells were cultured in DMEM supplemented with 10% foetal calf serum. One day prior to the transfection, the cells were plated onto 12-well plates at a density of 80,000/well. The next day, the cells were co-transfected with the pGl3 constructs, the internal control vector pRL-TK (Thimidine Kinase promoter-Renilla luciferase report luciferase; (Promega, Madison, WI, USA) and Lipofectamine 2000 (The cells were cultured for 4 h in DMEM without serum and antibiotics. Afterwards, the medium was changed, and the cells were incubated for further 24 h

C2C12 cells were cultured in DMEM supplemented with 10% foetal calf serum. Aliquots of 70,000 cells were co-transfected with the pGl3 constructs, specific expression vectors, renilla report internal control and Lipofectamine 3000, then plated on 24-wells and cultured in DMEM supplemented with serum for 48 h.

At the end of the incubation times, cells were washed with PBS and then 100 μL of lysis buffer (Promega, Madison, WI, USA) was added to the wells. The cell lysates were collected and centrifuged for 5 min at 12,000× *g*. Aliquots of 30 uL were assayed for luciferase activity by using the Dual-Luciferase Reporter Assay kit (Promega, Madison, WI, USA) and measured by the GloMax luminometer (Promega, Madison, WI, USA). The following software: Trap database, Tfsitescan, Animal TF database, and Transfact were utilized to analyze/identify the putative binding sites for transcription factors on PDE5 promoters.

### 4.7. Statistical Analysis

The data are presented as the mean ± standard deviation (SD). Statistical significance between groups was determined using GraphPad Prism 5.0 software. *p* values of less than 0.05 were considered statistically significant.

### 4.8. Bioinformatics

The following software: Trap database, Tfsitescan, Animal TF database, and Transfact were utilized to analyze/identify the putative binding sites for transcription factors on *Pde5a* promoters.

## Figures and Tables

**Table 1:** Primers used in RNA analysis and promoters cloning.

Gene	Forward Primer (5′-3′)	Reverse Primer (5′-3′)
Pde5a1	ATGGAACGAGCGGGCCCCAACT	GGTCAACTTCTGCATTGAA
Pde5a2	ATGTTGCCCTTTGGAGACAAAAC	GGTCAACTTCTGCATTGAA
Pde5a3	GTTTTGTTCTACAGAGACATG	GGTCAACTTCTGCATTGAA
Pde5a1 (qPCR)	GGACTGGGTGGAAGCGTGGCTG	GCAAGAGCAGGACTCGGTATGGGC
Pde5a2 (qPCR)	ATGTTGCCCTTTGGAGACAAAACG	GCAAGAGCAGGACTCGGTATGGGC
Gapdh	TCGTCCCGTAGACAAAATGG	TTGAGGTCAATGAAGGGGTC
Pde5a1-1	ggtaccAACCACTCAGCCAGAATGAAGT	aagcttCGTCCCTGCAGTGTCTCAGCG
Pde5a1-2	ggtaccCCACCAAGAGTTGCTTA	aagcttGAGTTGGGGCCCGCTCGTTC
Pde5a1-3	ggtaccCCACCAAGAGTTGCTTA	aagcttGAGTTGGGGCCCGCTCGTTC
Pde5a2-1	agatctAGAAGAGGAGCCACAAAGGACACAG	aagcttCCAAAGGGCAACATAGCAAAG
Pde5a2-2	agatctGGAGCTTTGCGGCGCGCACACAAA	aagcttCCAAAGGGCAACATAGCAAAG
MyoD	CCCCGGCGGCAGAATGGCTACG	GGTCTGGGTTCCCTGTTCTGTGT
Runx1	CGGCCATGAAGAACCAGGTA	CAACTTGTGGCGGATTTGT

## Data Availability

Promoter sequences have been deposited in the NCBI database (accession number, PDE5A1: KR816337; PDE5A2: MG672019).

## Supplementary Materials

The following supporting information can be downloaded at: https://www.mdpi.com/article/10.3390/ijms24010703/s1.

## Abbreviations

PDE: Phosphodiesterase; cAMP: Cyclic adenosine monophosphate; cGMP: Cyclic guanosine monophosphate; STZ: streptozotocin; MyoD: myogenic differentiation protein 1; Runx1: Runt-related transcription factor 1; Atf3: activating transcription factor 3; Egr1: early growth response 1.
