# Supplementary material for: Phosphodiesterase 5a Signalling in Skeletal Muscle Pathophysiology
Source: Int J Mol Sci. 2022 Dec 31;24(1):703. doi: 10.3390/ijms24010703 (PMC9820699; doi:10.3390/ijms24010703)
Supplement: Supplementary file 1 [file ijms-24-00703-s001.zip › ijms-2074683-supplementary.pdf]

Supplementary Figure S1. Immunofluorescence staining of Pde5a in proliferating (C_2C_{12} myoblasts) and differentiating mouse myogenic cells (C_2C_{12} myotubes). Green: Pde5; red: actin. Nuclei are stained with DAPI (blue).

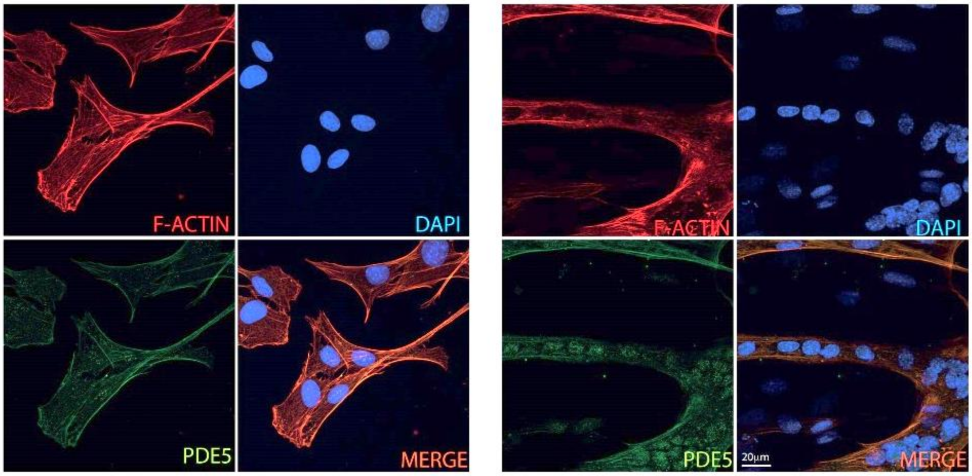

C_2C_{12} myoblasts

C_2C_{12} myotubes

Supplementary Figure S2.
a) Schematic representation of *Pde5a* gene showing the localization of the two regions cloned and assayed for promoter activity; b) sequences of the regions cloned in luciferase vectors; c) aminoacid sequence of the first 2 exons of Pde5a murine variants showing that these isoforms differ in the N-terminal region.

a)

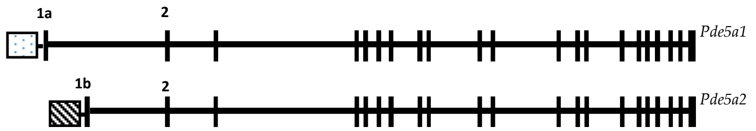

b)

5′ gene region

Pde5a1-1 promoter

aaccactcagccagaatgaagtgtcaatatgaactttggagggagcgggcacaaacagttaaggcccaatttctagctat
tatcccagagttcagttttaaatcaggtgacttcctggtaggaggaatgtgtagatatggggtgggggtggggttcacca
acaaggggaaactccctattcgctaagcatatttaatgtcatagtaacatatcaggagagaactgtcattttaaaatcta
atataatttatcatgtgttgctttggtcatggaattttatcacagcaataagcaatgtaaggcaatactatttctgaggt
atcgatgccagttaaatacaaatgagttcattttaaatcaggctaactgggtgttcccagttgtacatgggtagctagtg
ttagggttcgtaagtcagcagaatcgtgtagaggtagtcttagcttcaaaattcacaggccaaggacaggtggttgtcat
ttttatgtcctatgtctaaccgaatcttgcgcttccataaggagggtctttggtgtatgaatcaatgattacaactgatg
ttagccttaagctcggatgtggttttgatggaagccactgtgtcccgtacctgcaggtttgtcatagcggtttcttcctc
atgtctctccctagcttgtagggttggagtgctttccggagtcactgggagggctgctgtgagggagataggttctgggt
cctctccaccaagagttgcttaaggagaaaagtgaggtggggctgtccctgtactactaagaacgaagaaaaacaatcac
gttaactttttcccacactttctagtgaagctcctgcctcttgccttctggactgtagatgagtcctcactgtgatcttt
ctgtcctagagttggttggttgggaattgcaaatccacccactgtcacctttgcgcatagagggaagctgactcggccca
cagcgcaccccagcggcggtctggagtccttctagccttggccacttggcttccggagggctcgccagtcagcgccgagg
cagatgcagaaagccggtttccgcgggaccccaaggaggacgcccgccacctgggaacctcggggacctggagggggcggg
gctgagcgagctccggggccggcccgcgtctctgtggcttcgctgagacactgcagggacgaataaaggcaaaaggggag
ggtctcgaacactgtcctgctcctctgagagagagaggtggaaatccggcacctccgagccgcagagacgcgcggcaagc
acc***ATGGAACGAGCGGGCCCCAACTCCGTGCGGTCGCAGCAGCAGCGGGACCCGGACTGGGTGGAAGCGTGGCTGGATGA***
TCACCGGGACTTTACCTTCTCTTACTTTATTAGAAAGGCCACCAGGtaagaagaggagccacaaaggacacagccaggga

Exon 1a

intron I

Pde5a2-1 promoter

catgaagcgtgacctgaggctgatctctctcccccaccattgaattttcccctcgatccaacattaaacaatccccgttc
ccaggccctttgcttcggaagtagcttctcggtcccagtacggactggagtcctctacttaaagggagcacagttgagaa
gcgaggcgtcggattagaaaggacttgcccacgaagttaaaaacaaaagcgtacacccggccctggagcaagattactct
cccgggccttctcccctcccctctactctcccggtttccctcccaaacccgcttagcaaaaaccaaaacaagccagagcc
ccttcccagcggctccggaagcaccactgcgggcgcgtccctgtggtcgtgctcctcccctgcccggcttgctctgaccc
tggagggggcggggaagccggggggtctggaccaggccaccgggaagcctgggtgccgcagcttgcccgggtcgcccccca
gtgaggactgtcgcctttggtcccccaatagtcctctggtcatccctcttctggctgcgtcgccgcaaggaggattttcg
cggagctttgcggcgcgcacacaaagggcgggagggaggcggcccgggcggactcggggacccggggacccggagcaccc
cagaggccagttcttcctggtctcgagccaaacgccgggaggccgctgcggagggcgtggagaagggagcctgtccttta
ggatccgactgctgcacgggcagtcggggctcgcgcagacttgagagtggagcggagggacgagagggtggagcggagtg
cgtggcgggcgggtcccccgaagctcgaggagtcgccgtgaagcccgcgcgcgcgcttgcctccggcgcgccgatctggg
ctgaactaaccagctctgctttggagaaggggctggagccccgccaccggcgccttggggcaacggccgtcgagcagctg
gtgctggccgcccgagccgggagggcgactctcgccgggcggggatggtggacgagccggtggggactcgcccgagcgcg
gccgccactcggccgggcgctgcccgccaggggcgacgcaacgcggggtccgcagccggcgacccgcagcgaagaggcg
gcgggggcagtaggggagccggtcgggcactcacggaactccgtcggcccccagagtctcccgagtccaccatccgagga
gttcgcaaacgagcgccccacctttgct***ATGTTGCCCTTTGGAGACAAAACGAGG***tacttccttcaacatcccg......
.... intron II (~ 17160 bp)..............................

Exon 1b

cttccttcaagtatttaagatgtctacatgttttgttctacagagac***ATGGTCAACGCATGGTTTTCAGAGAGAGTTCAC***
AACATCCCTGTGTGCAAGGAAGGCATCCGAGCCCATACCGAGTCCTGCTCTTGCTCTCTGCAGCAAAGTCCTCATGCAGA
TAATACCACCCCTGGAGCACCAGCCAGAAAAATATCTGCCTCTGAATTTGACCGACCTCTTAGACCCATTGTTGTCAAGG
ATTCCGAGGGAACAGTGAGCTTTCTCTCTGACTCAGGAAAAAAGGAACAAATGCCACTAACCCCCCCAAGATTTGATAGT
GATGAAGGGGACCAGTGCTCAAGGCTCTTGGAGCTAGTAAAAGATATTTCCAGTCATTTGGATGTCACAGCATTGTGTCA
CAAAATTTTCTTGCATATCCATGGACTCATCTCTGCGGATCGCTATACCCTGTTCCTTGTCTGTGAGGACAGCTCTAAAG
ACAAATTCCTCATCAGCCGCCTCTTTGATGTTGCTGAAGGTTCAACATTGGAGGAAGCTTCAAATAACTGTATCCGTTTA
GAGTGGAACAAAGGCATTGTGGGACATGTGGCAGCTTTTGGCGAGCCCTTGAACATCAAAGATGCCTATGAG.......
.. intron III ...

Exon 2

c)

A1 **M**ERAGPNSVRSQQQRDPDWVEAWLDDHRDFTFSYFIRKATRDMVNAWFSERVHNIPV *Exon 1a*
A2 **M**LPFGDKTRDMVNAWFSERVHNIPV *Exon 1b*
A3 **M**VNAWFSERVHNIPV
CKEGIRAHTESCSCSLQQSPHADNTTPGAPARKISASEFDRPLRPIVVKDSEGTVSF *Exon 2*
LSDSGKKEQMPLTPPRFDSDEGDQCSRLLELVKDISSHLDVTALCHKIFLHIHGLIS
ADRYSLFLVCEDSSKDKFLISRLFDVAEGSTLEEASNNCIRLEWNKGIVGHVAAFGE
PLNIKDAYE...
